# Urine YKL-40 is associated with progressive acute kidney injury or death in hospitalized patients

**DOI:** 10.1186/1471-2369-15-133

**Published:** 2014-08-15

**Authors:** Isaac E Hall, Edward P Stern, Lloyd G Cantley, Jack A Elias, Chirag R Parikh

**Affiliations:** 1 Section of Nephrology, Department of Internal Medicine, Yale School of Medicine, New Haven, CT, USA; 2 Program of Applied Translational Research, Yale School of Medicine, 60 Temple Street, 6th Floor, Suite 6C, New Haven, CT 06510, USA; 3 Centre for Nephrology, University College London/Royal Free London NHS Foundation Trust, London, UK; 4 Division of Biology and Medicine, Brown University, Providence, RI, USA

**Keywords:** Acute kidney injury, Biomarker, BRP-39, Chitinase 3-like-1, Net reclassification improvement, YKL-40

## Abstract

**Background:**

A translational study in renal transplantation suggested YKL-40, a chitinase 3-like-1 gene product, plays an important role in acute kidney injury (AKI) and repair, but data are lacking about this protein in urine from native human kidneys.

**Methods:**

This is an ancillary study to a single-center, prospective observational cohort of patients with clinically-defined AKI according to AKI Network serum creatinine criteria. We determined the association of YKL -40 ≥ 5 ng/ml, alone or combined with neutrophil gelatinase-associated lipocalin (NGAL), in urine collected on the first day of AKI with a clinically important composite outcome (progression to higher AKI stage and/or in-hospital death).

**Results:**

YKL-40 was detectable in all 249 patients, but urinary concentrations were considerably lower than in previously measured deceased-donor kidney transplant recipients. Seventy-two patients (29%) progressed or died in-hospital, and YKL-40 ≥ 5 ng/ml had an adjusted odds ratio (95% confidence interval) for the outcome of 3.4 (1.5-7.7). The addition of YKL-40 to a clinical model for predicting the outcome resulted in a continuous net reclassification improvement of 29% (P = 0.04). In patients at high risk for the outcome based on NGAL concentrations in the upper quartile, YKL-40 further partitioned the cohort into moderate-risk and very high-risk groups.

**Conclusions:**

Urine YKL-40 is associated with AKI progression and/or death in hospitalized patients and improves clinically determined risk reclassification. Combining YKL-40 with other AKI biomarkers like NGAL may further delineate progression risk, though additional studies are needed to determine whether YKL-40 has general applicability and to define its association with longer-term outcomes in AKI.

## Background

Acute kidney injury (AKI) is common and appears to be increasing among hospitalized patients [1-3]. Observational studies demonstrate that even transient AKI is associated with adverse events ranging from increased hospital length of stay, to the development of end-stage renal disease and death [4-6]. However, the biological mechanisms that underlie the relationship between episodes of transient AKI and outcomes are not well understood.

In patients traditionally categorized as having “pre-renal” AKI, temporary increases in serum creatinine (SCr) concentrations are caused by changes in fluid status and/or renal perfusion. When there is no structural kidney damage in these cases, the diagnosis of AKI may not be causally associated with adverse outcomes. Conversely, a subgroup of patients within the current AKI classification scheme have structural kidney injury and are likely to be at higher risk of significant outcomes directly attributable to their renal diagnosis. Inability to discriminate such a group may have contributed to the lack of success in developing treatments for AKI [7].

We have evaluated the potential diagnostic and prognostic roles of several protein biomarkers measured from blood and urine in different clinical AKI settings [8-11]. One of the most promising biomarkers from our work and others’ has been neutrophil gelatinase-associated lipocalin (NGAL), a 25 kDa protein expressed at low levels by several cell types but at particularly high levels by cells in the distal nephron during periods of structural kidney injury [12]. While actively examining biomarkers like NGAL, we have also continued to investigate the potential clinical utility for other proteins not previously described in the context of AKI. As such, our group recently showed that the products of the chitinase 3-like-1 gene (called BRP-39 in the mouse and YKL-40 in man) modulate renal repair mechanisms after ischemic kidney injury in mice and can act as effective markers of renal injury in the setting of kidney transplantation in man [13]. By this model, YKL-40 levels should be low in pre-renal AKI but relatively high when overt structural kidney injury triggers repair mechanisms and contributes to clinical outcomes.

Pepe and colleagues described 5 phases of biomarker development for detecting cancer [14]; however, the same categories can be applied to biomarker studies of kidney injury and repair. Accordingly, in this phase 3 biomarker development and proof-of-concept study, we investigated whether urine YKL-40 on the day of AKI diagnosis from any cause can help predict outcome in hospitalized patients. In mouse models, BRP-39 does not rise until 24 hours after a renal insult and peaks on day 3. The delay from renal insult to clinical AKI diagnosis by SCr criteria is typically more than 24 hours, so we postulated that YKL-40 levels could already be discriminatory in urine collected on the day of AKI diagnosis. We also hypothesized that further prognostic information would be provided by a model that incorporates urine levels of both YKL-40 and NGAL, the most predictive AKI biomarker previously measured in this mixed cohort of hospitalized patients.

## Methods

This is an ancillary study to the previously described hospitalized AKI cohort [8]. Briefly, we prospectively screened all patients aged at least 18 years at Yale-New Haven Hospital between 2008 and 2009 for AKI Network SCr criteria (urine output not used) [15]. Patients were eligible if admitted with at least stage 1 AKI (using outpatient SCr as baseline) or developed at least stage 1 AKI during the hospitalization. We excluded patients with end-stage kidney disease or kidney transplant, those discharged within 24 hours of enrollment, and those with stage 3 AKI at enrollment (as we wanted to detect progression from mild/moderate AKI to more severe outcomes). AKI stage on the day of diagnosis and peak AKI stage during the admission were determined relative to baseline SCr: stage 1, increase in SCr by ≥0.3 mg/dl or 0.5 to <2-fold increase; stage 2, 2 to <3-fold increase; and stage 3, ≥3-fold increase, or SCr ≥4.0 mg/dl after a rise of at least 0.5 mg/dl, or acute dialysis requirement.

Baseline glomerular filtration rate (GFR) was estimated from baseline SCr using the 4-variable Modification of Diet in Renal Disease study equation [16]. Other patient characteristics (including comorbidities) were recorded from the medical histories obtained by admitting/consulting physicians. For descriptive purposes, AKI type was determined by retrospective chart adjudication as previously described [8]. Study physicians considered all available notes and hospital data (e.g., nephrology consult notes and urine electrolytes when available, changes in creatinine and urine output, timing and amount of fluids given, patient disposition, etc.) to classify AKI as acute tubular necrosis (ATN), pre-renal azotemia, or “other”. The primary outcome was a composite of worsened AKI Network stage or in-hospital death. For further details, see our previous publication from this cohort.

We adhered to the Declaration of Helsinki in conducting this study, which was approved by the Yale Institutional Review Board (IRB). Waiver of written consent allowed for the immediate collection of urine (that would have otherwise been discarded) in real time from any patient that met inclusion criteria along with their de-identified hospital information and pre-admission baseline SCr.

### Specimen handling and biomarker measurement

One 10-ml urine sample was collected from the catheter tubing, or a clean catch was requested directly from the patient within a few hours of the routine morning blood draw that indicated the patient had AKI. Samples were immediately refrigerated and then centrifuged at 5000 × g for 10 minutes at 4°C within 4 hours of collection. Samples were stored in separated, 1 mL aliquots without additives at -80°C till biomarker measurements. Urine YKL-40 levels were assessed in the last quarter of 2012 using enzyme-linked immunosorbent assay (ELISA) kits as previously described (Quidel, San Diego, CA) [13]. Population based urine levels have not been defined for YKL-40, but published data for 22 non-diabetic patients with normal renal function indicate urine YKL-40 is typically undetectable or less than 0.2 ng/ml in healthy individuals [17]. Urine NGAL levels were measured via ELISA by the Devarajan laboratory between 2008 and 2009 as reported in our prior publication [8]. Population mean levels of urine NGAL in healthy adults are between 10–23 ng/ml [18]. Note, the documented intra-assay and inter-assay coefficients of variation for both YKL-40 and NGAL ELISA methods are less than 10%. All laboratory measurements were performed by personnel blinded to patient information.

### Statistical analysis

Our previously described baseline clinical model for predicting risk of the primary outcome included the following variables: age ≥65 years, body mass index, male gender, non-White race, baseline GFR, diabetes, hypertension and surgery before AKI. We used a predefined cutoff of ≥5 ng/ml for all analyses based on the median urine YKL-40 value for recipients with immediate graft function in previously published results from our kidney transplant cohort [13]. We used multivariate logistic regression, incorporating variables in the baseline clinical model, to calculate odds ratios (95% confidence interval) for the primary outcome according to YKL-40 value. We also determined the continuous net reclassification index (cNRI) and integrated discrimination index (IDI) as suggested by Pencina and colleagues [19,20].

We performed secondary analyses combining urine YKL-40 with our previously reported results for urine NGAL after determining the Pearson correlation coefficient for these biomarkers. We first divided the cohort by the upper quartile NGAL value (i.e., above 235 ng/ml), representing the most severely injured group according to this biomarker, and then subdivided the upper NGAL quartile by YKL-40 above or below 5 ng/ml. We compared primary outcome rates in the three non-overlapping groups using chi-squared tests. We also evaluated the distribution of adjudicated AKI type according to this biomarker pattern. Based on our preclinical understanding that YKL-40 is released in the setting of severe injury [13], we performed this simple step-wise approach for combining biomarkers to explore the potential for added information with YKL-40. We subsequently compared median (interquartile range) urine levels for both YKL-40 and NGAL based on the presence or absence of key baseline clinical characteristics using Mann–Whitney U tests. All analyses were performed with SAS version 9.3 for Windows (SAS Institute, Cary, NC). A Type I error of 0.05 (two-tailed) was considered statistically significant for all analyses.

## Results

### Cohort description and YKL-40 measurements

A total of 284 hospitalized patients with AKI were enrolled in the study, and after exclusions (see previous description), 249 were available for analysis. Seventy-two patients (29%) developed the primary outcome (progression of AKI stage or in-hospital death). Baseline characteristics are summarized in Table 1. Median urine levels for both YKL-40 and NGAL separated by these baseline characteristics are provided in Table 2.

**Table 1:** Baseline characteristics and outcomes by urine YKL-40 value

		**Urine YKL-40**		
**Characteristic**^**1**^	**All (N = 249)**	**<5 ng/ml (N = 218)**	**≥5 ng/ml (N = 31)**	**P-value**^**2**^
Demographics				
Age ≥65 years	143 (57)	121 (56)	22 (71)	0.10
Male sex	143 (57)	125 (57)	18 (58)	0.94
Non-White race	59 (24)	52 (24)	7 (23)	0.88
Body Mass Index, kg/m^2^	29.9 ± 8	30 ± 8	27 ± 6	0.06
Clinical Characteristics				
Pre-existing CKD	62 (25)	54 (25)	8 (27)	0.87
Pre-existing proteinuria	63 (32)	49 (29)	14 (50)	**0.03**
ACEi/ARB at enrollment	88 (36)	78 (37)	10 (34)	0.82
Hypertension	171 (69)	147 (68)	24 (77)	0.28
Diabetes	102 (41)	88 (41)	14 (45)	0.64
CHF	92 (37)	83 (39)	9 (29)	0.30
CAD	102 (41)	88 (41)	14 (47)	0.52
Stroke	34 (14)	26 (12)	8 (26)	**0.04**
Liver failure/Cirrhosis	27 (11)	24 (11)	3 (10)	0.82
Active cancer	60 (24)	46 (21)	14 (45)	**0.004**
# of Comorbidities				0.31
None	17 (7)	16 (8)	1 (3)	
1	34 (14)	31 (15)	2 (7)	
≥2	198 (79)	160 (77)	26 (90)	
Tobacco use				0.10
Never	130 (52)	118 (58)	12 (43)	
Prior	60 (24)	48 (24)	12 (43)	
Current	42 (17)	38 (19)	4 (14)	
Enrollment location				0.12
ICU	120 (48)	101 (46)	19 (61)	
Floor	129 (52)	117 (54)	12 (39)	
Renal Function				
Baseline SCr, mg/dl	1.2 ± 0.5	1.2 ± 0.5	1.2 ± 0.5	0.44
Baseline GFR^3^	68.5 ± 30	68 ± 30	72 ± 28	0.49
Stage of AKI at enrollment				**0.001**
Stage 1	207 (83)	188 (86)	19 (61)	
Stage 2	42 (17)	30 (14)	12 (39)	
Stage of AKI at peak SCr				**0.006**
Stage 1	176 (71)	162 (74)	14 (45)	
Stage 2	41 (16)	33 (15)	8 (26)	
Stage 3	18 (7)	13 (6)	5 (16)	
Stage 3-Dialysis^4^	14 (6)	10 (5)	4 (13)	
AKI type				
ATN	51 (20)	40 (18)	11 (35)	**0.03**
Pre-renal	164 (66)	151 (69)	13 (42)	**0.003**
Other	34 (14)	27 (12)	7 (23)	0.12
Discharge SCr, mg/dL	1.4 ± 0.7	1.4 ± 0.7	1.3 ± 0.5	0.34

Values are *n* (%) or mean ± SD. AKI, acute kidney injury; ATN, acute tubular necrosis; CKD, chronic kidney disease (if specifically listed in patient medical history); ACEi/ARB, angiotensin-converting enzyme inhibitor or angiotensin II receptor blocker medications; CHF, congestive heart failure; CAD, coronary artery disease; ICU, intensive care unit; SCr, serum creatinine.

^1^Data for the presence/absence of the characteristics listed were missing in no more than 5 patients each except for baseline proteinuria (missing for 51 patients) and ACEi/ARB use (missing for 7 patients). The values or percentages shown for each characteristics account for non-missing data only.

^2^Chi-squared or t-test. Values in boldface are significant at P<0.05.

^3^GFR (in ml/min per 1.73 m^2^) was estimated by the 4-variable Modification of Diet in Renal Disease equation.

^4^Those acutely dialyzed for AKI; excludes those with stage 3 AKI who were not dialyzed.

**Table 2:** Urine YKL-40 and NGAL values by baseline characteristics

**Clinical characteristics**	**YKL-40, ng/ml**			**NGAL, ng/ml**	
	**N**	**Median (IQR)**	**P-value**^**1**^	**Median (IQR)**	**P-value**^**1**^
Pre-existing CKD					
No	182	0.26 (0.08-0.85)	**0.001**	62 (19–237)	0.47
Yes	62	0.08 (0.03-0.31)		60 (16–192)	
Pre-existing proteinuria					
No	135	0.25 (0.07-0.59)	0.25	49 (16–235)	**0.004**
Yes	63	0.27 (0.07-2.33)		125 (44–388)	
ACEi/ARB at enrollment					
No	154	0.25 (0.08-1.05)	**0.03**	92 (24–298)	**0.02**
Yes	88	0.10 (0.06-0.54)		42 (17–141)	
Hypertension					
No	77	0.21 (0.08-0.68)	0.66	77 (20–249)	0.58
Yes	171	0.18 (0.06-0.97)		58 (18–234)	
Diabetes					
No	145	0.18 (0.05-0.66)	0.34	54 (18–227)	0.97
Yes	102	0.22 (0.07-1.41)		67 (18–235)	
CHF					
No	154	0.27 (0.07-1.41)	**0.02**	80 (21–336)	**0.05**
Yes	92	0.13 (0.05-0.54)		47 (15–141)	
CAD					
No	145	0.25 (0.07-0.93)	0.26	99 (27–323)	**0.003**
Yes	102	0.17 (0.06-0.71)		41 (14–121)	
Stroke					
No	213	0.18 (0.06-0.81)	0.28	54 (19–210)	0.43
Yes	34	0.24 (0.06-2.63)		102 (15–365)	
Liver failure/Cirrhosis					
No	222	0.18 (0.06-0.82)	0.26	53 (18–187)	**0.04**
Yes	27	0.30 (0.10-1.05)		237 (21–791)	
Active cancer					
No	188	0.17 (0.06-0.63)	0.07	44 (14–165)	**0.001**
Yes	60	0.29 (0.06-2.48)		164 (44–416)	

IQR, interquartile range; CKD, chronic kidney disease (if specifically listed in patient medical history); ACEi/ARB, angiotensin-converting enzyme inhibitor or angiotensin II receptor blocker medications; CHF, congestive heart failure; CAD, coronary artery disease.

^1^Mann–Whitney *U* test. Values in boldface are significant at P<0.05.

**Figure 1:**
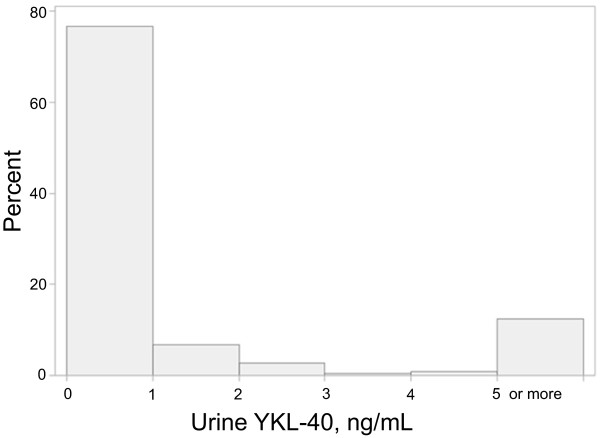
**Distribution of urine YKL-40.** YKL-40 ≥ 5 ng/ml was set at 5 ng/ml for this histogram. The 10^th^, 50^th^ and 90^th^ percentile values were 0.02, 0.2 and 13 ng/ml, respectively.

The median (10^th^-90^th^ percentile) value for urine YKL-40 was 0.2 (0.02-13) ng/ml. Our prior study demonstrated that recipients with the most rapid improvement in renal function immediately after kidney transplant had median urinary YKL-40 levels of 5 (0–60.7) ng/ml [13]. Eighty-eight percent of the current cohort had urinary levels below 5 ng/ml (Figure 1), and of those, 69% were adjudicated as pre-renal. In contrast, only 42% of those with YKL-40 ≥ 5 ng/ml were classified as pre-renal (P = 0.003).

**Figure 2:**
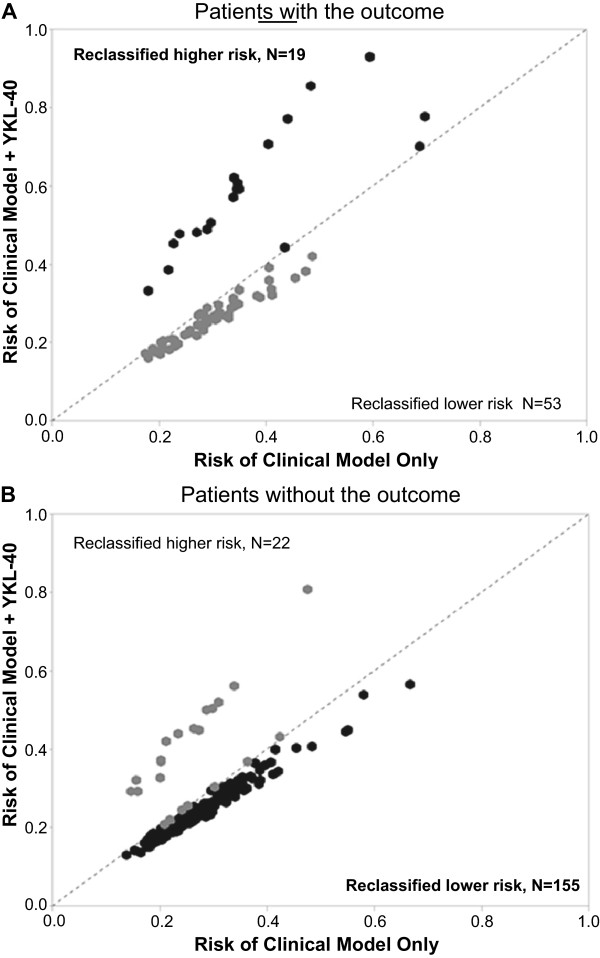
**Predicted risk using the clinical model plus urine YKL-40 versus the clinical model alone.** Each patient has a predicted risk for the outcome determined by their individual factors in the clinical (regression) model. Adding YKL-40 to the model results in a new predicted risk for each patient based on their YKL-40 value (above or below 5 ng/ml). For each patient, the predicted risk from the clinical model alone is plotted (horizontal axis) against the new predicted risk (vertical axis) after adding YKL-40 to the model. The dotted line represents no change in predicted risk (unity) after adding YKL-40. Changes in predicted risk can be considered appropriate or inappropriate based on the true outcome status. For example, in patients that ultimately developed the outcome **(A)**, increasing predicted risk would be appropriate and decreasing predicted risk would be inappropriate. The opposite is true for patients that did not develop the outcome **(B)**–decreasing predicted risk would be appropriate and increasing predicted risk would be inappropriate. Darker symbols here indicate patients that were reclassified in the appropriate direction.

Of the 31 patients with YKL-40 ≥ 5 ng/ml, 16 (52%) had the primary outcome compared to 56/218 (26%) of those with YKL-40 < 5 ng/ml (P = 0.003). For the individual components of the primary outcome, 12/31 (39%) versus 34/218 (16%) had progression of AKI stage (P = 0.002), and 10/31 (32%) versus 35/218 (16%) died in-hospital (P = 0.03), respectively.In regression analyses, the unadjusted odds ratio for the primary outcome with YKL-40 ≥ 5 ng/ml was 3.1 (1.4-6.6), and after adjusting for the variables in the clinical model, the odds ratio increased slightly to 3.4 (1.5-7.7). Our pre-defined cutoff for YKL-40 also significantly improved risk reclassification, particularly for patients that did not develop the outcome (Figure 2). Adding YKL-40 ≥ 5 ng/ml to the clinical model resulted in a cNRI of 29% (2.0%-56%, p = 0.04) and an IDI of 2% (0.04%-4.0%, P = 0.03).

### Injury-repair biomarker combination

**Figure 3:**
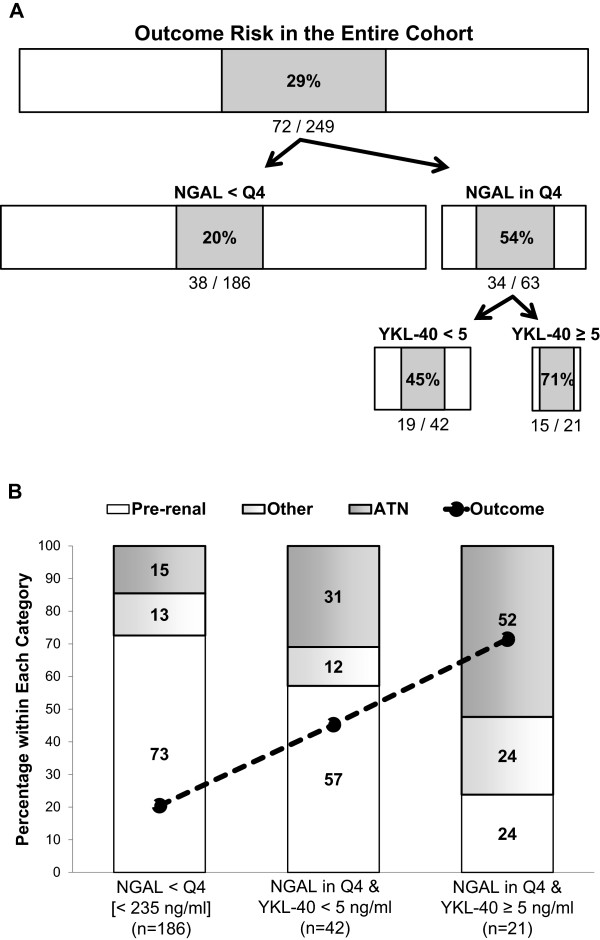
**Cohort partitioning by urine biomarkers with outcome risk and adjudicated acute kidney injury type. (A)** The cohort was partitioned into those with urine neutrophil gelatinase-associated lipocalin (NGAL) values in the fourth quartile (Q4, ≥235 ng/ml) and those in the first three quartiles. The former group was then separated into those with urine YKL-40 ≥ 5 ng/ml and <5 ng/ml, respectively. The percentage of patients within each group/node that experienced the primary outcome is shown, with horizontal bar widths that are proportional to the numbers of patients in that node. **(B)** The percentage of patients within each terminal node that developed the primary outcome is depicted here as a (dotted) line graph, and the distribution of adjudicated acute kidney injury type within each terminal node is depicted as a stacked bar graph with corresponding percentages as shown. ATN, acute tubular necrosis.

There was statistically significant, though fairly modest correlation between NGAL and YKL-40 in this cohort (r = 0.47, P < 0.001). The step-wise combination of NGAL and YKL-40 produced a straightforward algorithm for determining outcome risk (Figure 3A). The first group, with NGAL values below the fourth quartile, had the lowest risk of developing the outcome at 20%. The outcome rate in the first group was significantly different from the rates in both the second and the third groups—NGAL in the fourth quartile subdivided by YKL-40 less than or ≥5 ng/ml, respectively (P < 0.001 for both comparisons). The outcome rates were also statistically different between the second and third groups at 45% and 71%, respectively (P = 0.05). The same three-group injury-repair biomarker pattern also corresponded with AKI type with an increasing proportion of patients adjudicated as ATN and a decreasing proportion adjudicated as pre-renal (overall chi-square P < 0.001, Figure 3B). Of note, only 10 patients in the first group, with NGAL values below the fourth quartile, also had YKL-40 values ≥5 ng/ml, and only one of these patients developed the primary outcome (in-hospital death, no progression in AKI stage).

## Discussion

This is the first investigation of YKL-40 in urine from injured native human kidneys. Our findings support the hypothesis that urine YKL-40, a “repair-phase” protein, may have utility as a marker of severe kidney injury. The current cohort was more heterogeneous and likely had lower overall structural AKI severity than the cohort of deceased-donor kidney transplants we previously evaluated [13]. In keeping with this, we found urine YKL-40 in hospitalized patients with AKI of any cause to be detectable, but with levels that were markedly lower than those seen in peri-operative kidney transplant recipients. Our analyses also show the potential clinical benefit of considering a biomarker cutoff based on prior experience in complementary settings. We found a modest association between high levels of YKL-40 and AKI progression or death—patients with YKL-40 ≥ 5 ng/ml had a three-fold increase in the odds of this outcome.In addition, adding YKL-40 ≥ 5 ng/ml to our baseline clinical model significantly improved risk reclassification primarily for patients who would not develop the primary outcome. This improvement is clearly seen in Figure 2B, where overwhelmingly more patients without the outcome were correctly reclassified as lower predicted risk rather than higher predicted risk after adding YKL-40 to the clinical model. Furthermore, when combined in a simple step-wise approach with NGAL, the best injury biomarker previously measured in this cohort, YKL-40 ≥ 5 ng/ml significantly partitioned the high-risk group (defined by an NGAL value in the upper quartile) into moderate-risk and very high-risk groups. Taken together, these findings illustrate the concept that incorporating an additional biomarker, which has complementary mechanisms of action, can enhance the utility of other biomarkers or even improve upon clinical prediction models.

BRP-39/YKL-40 is a 39 kDa protein that lacks true chitinolytic activity, like all chitinase-like proteins, and is produced by several cell types including epithelial cells in the airway and colon, chondrocytes, hepatic stellate cells, vascular smooth muscle cells, fibroblasts and differentiated macrophages [21]. Our group recently provided preclinical and translational data regarding BRP-39/YKL-40 expression in the kidney, detectable urine levels, and the physiologic role it plays in limiting tubular cell apoptosis during the repair phase of AKI [13]. This work is an example of ongoing efforts to develop effective biomarker panels to assess renal health and prognosis in different clinical settings. As a proof of concept, the current study suggests that YKL-40 can be non-invasively measured in urine at the first clinical sign of AKI in general hospitalized patients (e.g., a rise in SCr or drop in urine output) and the results used in combination with other biomarkers (e.g., NGAL levels to quantitate distal nephron damage) in order to assess renal injury-repair processes in real-time and potentially improve outcome prediction.

While much AKI biomarker research has appropriately focused on diagnosing AKI earlier than SCr [22-25], there have been fewer advances in identifying novel biomarkers that can effectively classify patients with AKI as more likely versus less likely to progress [26,27]. A key application for a tool of this kind may lie in excluding patients at low risk of progression from enrollment into trials that evaluate management strategies initiated immediately after the diagnosis of AKI. Clinicians frequently use the term “pre-renal” AKI to describe these low-risk individuals, but the pre-renal state is typically assigned in retrospect after observing the patient’s response to a fluid challenge. Our current findings suggest that a straightforward combination of kidney injury-repair biomarkers may be useful for prospectively risk-stratifying potential trial subjects. To most effectively assess different treatment strategies in the context of a controlled trial, only participants with significant structural renal injury *and* at high risk for progression (e.g., high NGAL *and* high YKL-40 values) should potentially be randomized. As an aside but of particular interest to the AKI field, the misclassification of “pre-renal” patients as having structural AKI may also be diluting or otherwise complicating studies of novel biomarkers to diagnose AKI earlier than SCr [28].

There are some limitations to consider. First, this was an ancillary study to a prospectively collected observational cohort. Nonetheless, the cohort was assembled for the express purpose of evaluating the performance of traditional and novel biomarkers, like YKL-40, as they relate to in-hospital outcomes (i.e., to perform appropriate phase 3 biomarker development studies) [14]. In addition, we followed proper biorepository procedures including validated and blinded biomarker measurements from sample aliquots that had not undergone prior freeze-thaw cycles. The use of a dichotomous cutoff value for a continuous measurement can be considered a limitation; however, the value of 5 ng/ml for YKL-40 was pre-specified based on our previous findings in a separate patient cohort. In addition, sample size limited our ability to evaluate multiple biomarker combinations and comparisons. We therefore controlled for the same confounders and evaluated a simple, representative combination of YKL-40 with the best injury biomarker based on our prior analyses. Lastly, this study was limited by a lack of post-hospitalization information and outcomes given IRB approval with waiver of consent to study the effects of AKI on inpatient outcomes only.

## Conclusions

In summary, YKL-40 is a repair-phase protein that is detectable in urine on the first day of clinically apparent AKI and provides only modest prognostic potential on its own. Our findings suggest that there may be utility in combining YKL-40 with other biomarkers like NGAL to refine AKI prognosis and better determine the relationship between injury severity and the degree of repair activation. Further studies are needed to validate the performance of YKL-40 in a large population of patients with AKI and, in particular, to follow trends in this marker of renal injury and repair over the course of disease progression and/or recovery. The kidney is clearly an intricate organ with several cell types that are responsible for multiple homeostatic functions. As we continue to learn more about the complex mechanisms that maintain and repair the renal-urinary system following periods of stress and injury, we envision developing better clinical AKI biomarker panels that provide useful diagnostic and prognostic information relative to those mechanisms. With further insights into the renal responses to acute injury, novel biomarkers like YKL-40 may also begin to give us better information about overall kidney health and could suggest new therapeutic targets for study in humans.

## Abbreviations

AKI: Acute kidney injury; ATN: Acute tubular necrosis; cNRI: Continuous net reclassification index; GFR: Glomerular filtration rate; IDI: Integrated discrimination index; IRB: Institutional review board; NGAL: Neutrophil gelatinase-associated lipocalin; SCr: Serum creatinine.

## Competing interests

None of the authors have any competing interests to declare.

## Authors’ contributions

IEH participated in the design of the study, analyzed the data, interpreted the results and helped write the manuscript. EPS participated in the design of the study, interpreted the results and drafted the manuscript. LGC participated in the design of the study, interpreted the results and helped write the manuscript. JAE participated in the design of the study, was involved in carrying out the laboratory measurements for YKL-40, and helped write the manuscript. CRP conceived of the study, participated in its design, interpreted the results and helped write the manuscript. All authors read and approved the final manuscript.

## Authors’ information

Isaac E. Hall, MD, MS and Edward P. Stern, MBBS are co-first author.

## Pre-publication history

The pre-publication history for this paper can be accessed here:

http://www.biomedcentral.com/1471-2369/15/133/prepub
